# Using Different Methods to Access the Difficult Task of Delimiting Species in a Complex Neotropical Hyperdiverse Group

**DOI:** 10.1371/journal.pone.0135075

**Published:** 2015-09-02

**Authors:** Guilherme J. Costa-Silva, Mónica S. Rodriguez, Fábio F. Roxo, Fausto Foresti, Claudio Oliveira

**Affiliations:** 1 Universidade Estadual Paulista, Departamento de Morfologia, Laboratório de Biologia e Genética de Peixes, Botucatu, São Paulo State, Brazil; 2 Universidade Federal de Viçosa, Campus de Rio Paranaíba, Rio Paranaíba, Minas Gerais State, Brazil; University of Innsbruck, AUSTRIA

## Abstract

The genus *Rineloricaria* is a Neotropical freshwater fish group with a long and problematic taxonomic history, attributed to the large number of species and the pronounced similarity among them. In the present work, taxonomic information and different molecular approaches were used to identify species boundaries and characterize independent evolutionary units. We analyzed 228 samples assembled in 53 distinct morphospecies. A general mixed yule-coalescent (GMYC) analysis indicated the existence of 70 entities, while BOLD system analyses showed the existence of 56 distinct BINs. When we used a new proposed integrative taxonomy approach, mixing the results obtained by each analysis, we identified 73 OTUs. We suggest that *Rineloricaria* probably has some complexity in the known species and several species not formally described yet. Our data suggested that other hyperdiverse fish groups with wide distributions can be further split into many new evolutionary taxonomic units.

## Introduction

With the implementation of the Barcoding of Life project, the gene *COI* has been used for some time as a tool for identification of fish species. [1] This gene has been an efficient tool for delimiting species of particular taxonomic groups and providing evidence of independent evolutionary units or operational taxonomy units (OTUs) with the recognition of genetic patterns within groups that support the traditional taxonomic studies [2–4].

Barraclough et al. [5] suggested that many factors can affect the success rate of DNA barcoding, such as the typical levels of intraspecific and interspecific variation among clades and substitution rate variation among lineages, casting doubt on the power of this method to identify and delimit species. More recently, improved statistical methods have been proposed to analyze barcoding data that is being used to identify the "species boundaries" and thereby show the evolutionary independent units present in complex groups. One of the most popular approaches for species delimitation based on single-locus data is the general mixed yule-coalescent (GMYC), which is widely used in biodiversity assessments and phylogenetic community ecology [6–8]. This method identifies boundaries as a shift in branching rates on a phylogenetic tree that contains multiple species and populations. However, the GMYC analysis requires much computational time to identify large numbers of OTUs [9].

As an analysis that demands less computational time, the BOLD system employs the barcode index number (BIN) [9], and it is faster to run than the GMYC analysis. The BIN is the denomination given to the OTUs implemented in the BOLD system. Currently, the BOLD database contains more than 350,000 public BINs, flagging the existence of many new OTUs to be formally described. The BIN designation is a result of the refined single linkage (RESL), which is composed in two steps. The first step couples single linkage with a threshold (threshold = 2.2%). After this step, if one sequence is more divergent than two times the threshold (or 4.4%) of all sequences in the BOLD database, this sequence inaugurates a new BIN. Sequences with low genetic divergence (<4.4%) are submitted to the second step, which refines the search using Markov clustering that assigns sequences to a cluster and then to a new or a preexisting BIN (see more details in [9,10]). The BIN assignment is constantly updated, and, with implementation of sequence intermediates of two distinct BINs, they could therefore be merged.

According to Ratnasingham and Herbert [9], the GMYC and the BIN approach are very efficient for OTU identification, even in hyperdiverse groups. Several Neotropical fish groups (e.g., *Rineloricaria*) are considered hyperdiverse groups and have an old and problematic taxonomic history [11–13]. *Rineloricaria* is distributed throughout almost all basins of the Neotropical tropical region, from Panama to Argentina, occupying a broad variety of habitats [13,14]. In the last few decades, an increasing number of studies related to *Rineloricaria* have led to the description of 18 new species. Today, this genus has 65 valid species, but several species differed only subtly (e.g., *R*. *cadeae* and *R*. *longicauda*) [14,15], and the total number may be underestimated [16–18]. There are species with great morphological plasticity (e.g., a number of abdominal plates) such as *R*. *microlepidogaster* and *R*. *capitonia* [14,19]. Many species in *Rineloricaria* have high levels of intraspecific variation, which has led to difficulty delimiting species boundaries; consequently, the taxonomic advances within this genus are slow and are mainly related to the recognition of new species [20]. A broad characterization could be important for the delimitation of *Rineloricaria* species and may be helpful to the alpha taxonomy of this group. In the present study, we used single-locus DNA sequences of the *COI* gene and morphological information to delimit the species and discuss species boundaries in *Rineloricaria*.

## Material and Methods

### Ethical statement

We declare that the fish under study are not protected under wildlife conservation, and no experimentation was conducted on live specimens. All specimens used were collected in accordance with Brazilian laws, and the sampling was approved by the Brazilian Institute of Environment and Renewable Natural Resources (IBAMA) and Sistema de Autorização e Informação em Biodiversidade (SISBIO) under a license issued in the name of Dr. Claudio Oliveira (SISBIO number 13843–1). After collection, the animals were anesthetized and sacrificed using 1% benzocaine in water as approved by the Bioscience Institute/UNESP Ethics Committee on the Use of Animals (CEUA; protocol 405) and recommended by the National Council for the Control of Animal Experimentation and the Federal Board of Veterinary Medicine.

### Sampling and geographic distribution

This study was conducted with 228 specimens representing 38 nominal species (60.3% of all recognized species of Rineloricaria) and 15 possible new species (see S1 Table for sample data summary). Vouchers and tissues were deposited in the fish collection of the LBP (LBP- Institutional acronyms [21]), Departamento de Morfologia, Instituto de Biociências, UNESP, Botucatu, São Paulo State, Brazil.

### DNA Extraction and Sequencing

Total genomic DNA was isolated from fins or muscle tissues of each specimen with a DNeasy Tissue Kit (Qiagen), according to the manufacturer’s instructions. Amplifications were performed in a total volume of 12.5 μl, with 1.25 μl of 10X buffer (10 mM Tris-HCl+15 mM MgCl2), 0.5 μl dNTPs (200 nM of each), 0.5 μl each 5 mM primer (FishF1, FishR1 or FishF2, FishR2 described in [22], 0.25 U Platinum *Taq* Polymerase (Invitrogen), 1 μl template DNA (12 ng), and 8.7 μl ddH2O. The PCR reactions consisted of 30–40 cycles for 30 s at 95°C, 15–30 s at 48–54°C (according to each species), and 45 s at 72°C. All PCR products were first visually identified on a 1% agarose gel and then purified using ExoSap-IT (USB Corporation) following the instructions of the manufacturer. The purified PCR products were sequenced using a Big Dye Terminator v 3.1 Cycle Sequencing Ready Reaction Kit (Applied Biosystems), purified again by ethanol precipitation and loaded onto an automatic sequencer 3130-Genetic Analyzer (Applied Biosystems).

### Sequencing analysis

Consensus sequences from forward and reverse strands were obtained using Geneious Pro 5.4.2 [23]. To avoid analyzing sequences of nuclear mitochondrial pseudogenes (numts) we followedthe recommendations of Song et al. [24] and only the sequences that have gone through all quality steps were used to the analysis on present study. Alignments were generated using Muscle [25] under default parameters. After alignments, the matrix was checked by eye for any obvious misalignments and to detect potential cases of sequencing errors, and the presence of stop codons was checked using Geneious.

Nucleotide variation, substitution patterns and genetic distances were examined using the BOLD system tools. To evaluate the occurrence of substitution saturation, we estimated the Iss index in DAMBE 5.2.31 [26], as described by Xia et al. [27] and Xia and Lemey [28], and the rate of transitions/transversions was also evaluated with the software DAMBE 5.2.31. The best nucleotide evolution models for the *COI* gene were evaluated using Modeltest 3.06 [29] under the information-theoretical measure of Akaike Information Criterion (AICc).

### Integrative taxonomy of *Rineloricaria*

For species delimitation and consequent identification of the OTU, we used traditional morphological identification and two molecular analyses: the BIN and the GMYC model.

#### BIN identification

The BIN analysis was carried out automatically in the BOLD system. Each sample with a sequence longer than 500 bp was assembled in a preexisting BIN in the BOLD database or assigned as a new BIN (for more details about the attribution of a BIN performed in the BOLD system, see [9]).

### GMYC analyses

The lognormal relaxed molecular clock tree was estimated using BEAST v.1.6.2 [30] because the GMYC requires an ultrametric tree. The nucleotide evolutionary model used to estimate the ultrametric tree was the GTR model with a Gamma distribution (estimated by the program Modeltest 3.06). Briefly, we used Bayesian inference of phylogeny with a relaxed lognormal clock and birth-death speciation process rate on an arbitrary timescale. A random tree was used as a starting tree for the Markov chain Monte Carlo searches. Eight chains were run simultaneously for 10,000,000 generations, and a tree was sampled every 100th generation. The above analysis was performed twice. The distribution of log-likelihood scores was examined to determine the stationary phase for each search and to decide whether extra runs were required to achieve convergence using the program Tracer 1.6 [31]. All sampled topologies beneath the asymptote (2,500,000 generations) were discarded as part of a burn-in procedure, and the remaining trees were used to construct a 50% majority-rule consensus tree in TreeAnnotator v1.6.2.

We performed the GMYC analysis with R 3.0.0 [32] with a single threshold method with the Species Limits by Threshold Statistics (“splits”) package (http://r-forge.r-project.org/projects/splits) on standard parameters.

### Final OTU identification

**Fig 1:**
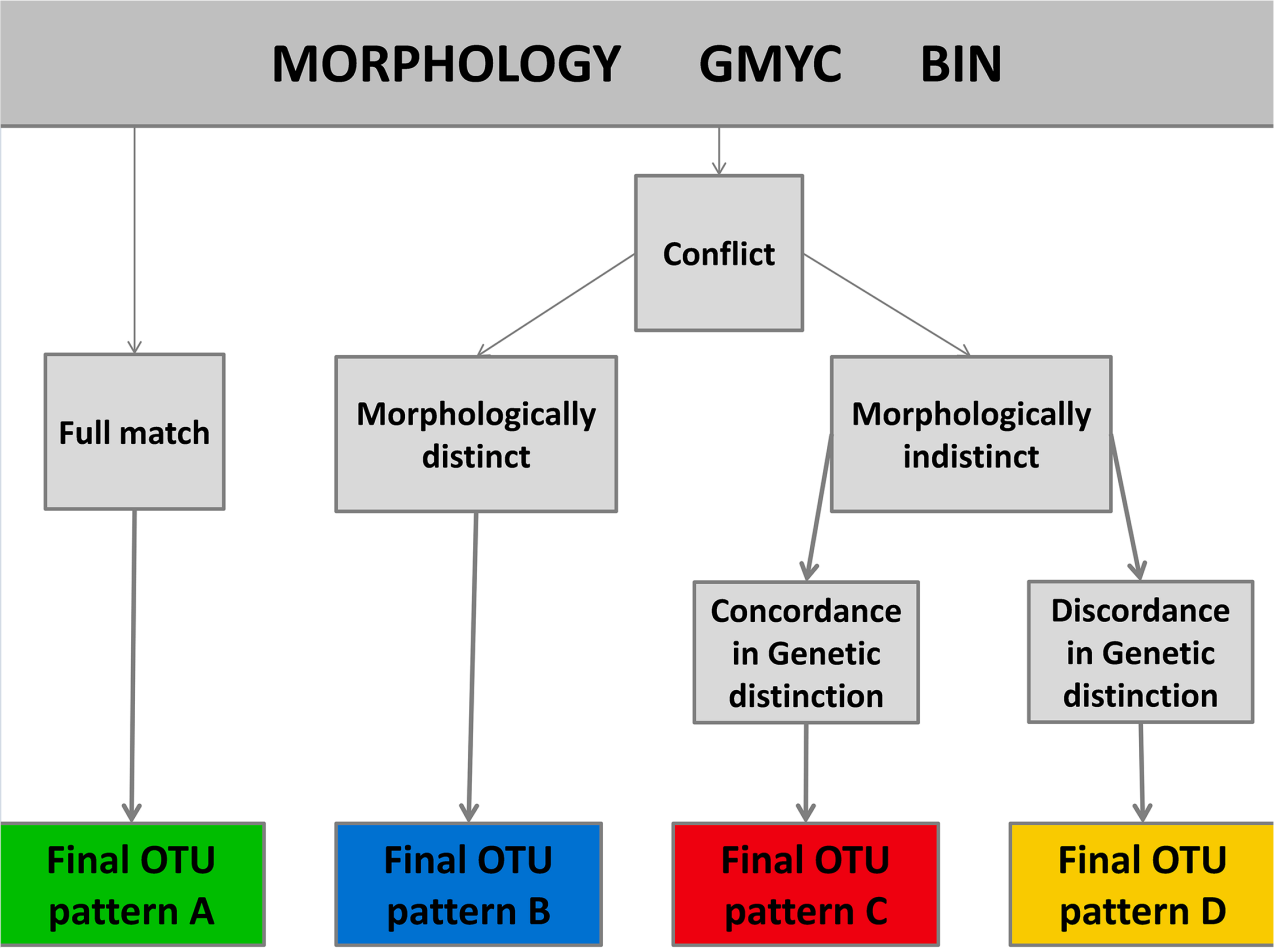
OTU definition criteria. When all methodologies were in accordance in distinguishing a sample (or sample group), we considered it as a single OTU of the pattern A. When the OTU was morphologically distinct but genetically indistinct (at least by one genetic methodology), we considered it as OTU of the pattern B. When the OTUs were genetically distinct (by GMYC and BIN) but morphologically indistinct, they were considered as distinct OTUs of the pattern C. When the OTU was distinct by only one genetic methodology, it was considered to be of the pattern D.

For the definition of OTUs, we followed an integrative approach using the GMYC model, BIN and traditional morphological identification. Here we considered OTU for groups that were distinct by at least one of the methodologies. However, we know that a hypothesis is more robust when supported by more than one methodology; in this way, the OTU definition hypothesis was categorized for distinct patterns, shown in Fig 1.

## Results

We obtained barcode sequences for 225 specimens with more than 500 base pairs (BOLD numbers: BRINE-1-14 BRINE-225-14). Stop codons, deletions or insertions were not observed in any sequence. After alignment and editing, the final matrix had 533 characters, of which 328 positions were conserved and 205 were variable, with 26.4% of adenine, 26.6% of cytosine, 30.9% of thymine and 16.1% of guanine. The data were not saturated, considering that the Iss.c value was greater than the Iss, and the R^2^ value was greater than 0.83 for transitions and transversions. The genetic distance analysis revealed that 96% of the morphospecies (monophyletic cluster of all named specimens) differed from each other by more than 2% of the Kimura 2 Parameter (K2P) distance, whereas some morphospecies had a high genetic variation (maximum 8.5%), and, sometimes, the variation was larger than the species divergences (minimum 0.8%; see more details in S2 Table and S1 Fig). Therefore, we did not observe a “barcoding gap” within *Rineloricaria* (see more in S1 Fig).

**Fig 2:**
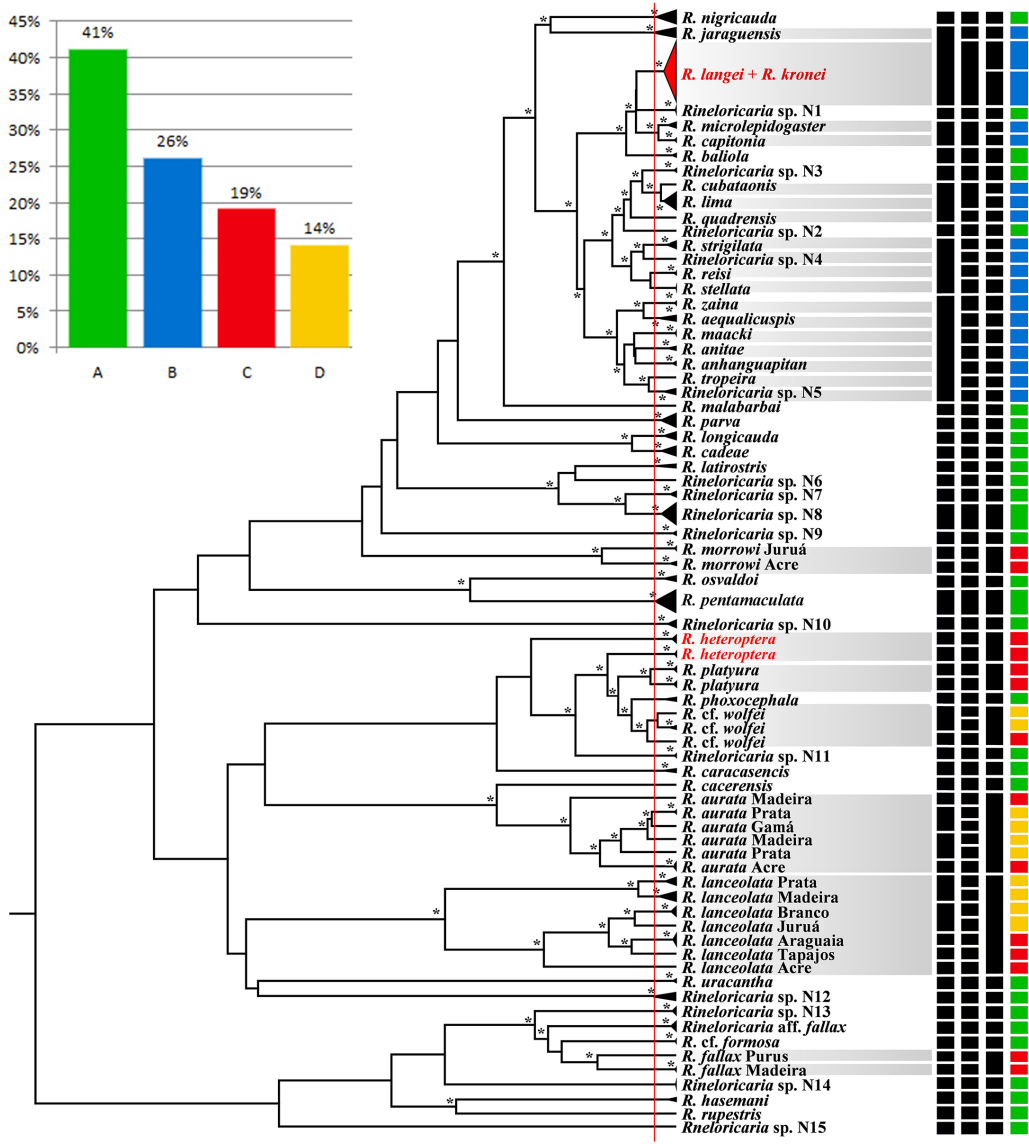
Bayesian phylogenetic tree of *Rineloricaria* obtained with *COI* data. The asterisks in the node branches represent a posterior probability higher than 95%. The vertical red line in the tree shows the transition point from Yule to a coalescent branching process in the analysis of all sequences, as estimated by the single-threshold model in the GMYC test. The species in the gray squares have questionable taxonomy. Red marks represent the clusters with morphospecies not reciprocally monophyletic. The first three black vertical columns represent, respectively, the status of the identification of OTUs by the BIN, GMYC and morphology criteria, while the fourth column represents the final OTUs proposed according to the criteria shown in Fig 1. The OTU color corresponds to the colors used in Fig 1

The phylogenetic analysis resulted in a tree with high statistical support for the terminal nodes and low support for the basal nodes (Fig 2). All morphospecies were monophyletic, except *R*. *heteroptera*, *R*. *kronei* and *R*. *langei*. *R*. *heteroptera* was recovered as paraphyletic with two distinct, well supported clades, while *R*. *kronei* and *R*. *langei* were not reciprocally monophyletic and, consequently, were phylogenetically indistinct by the *COI* marker.

The integrative taxonomy analysis found 73 OTUs following the criteria shown in Fig 1. Thirty OTUs were identified with all methodologies (Fig 1 pattern A), while 43 OTUs had interpretation conflicts. Among the 43 OTUs with identification conflicts, 19 were morphologically distinct (Fig 1 pattern B), 14 OTUs were distinguishable by both genetic methodologies (Fig 1 pattern C) and 10 OTUs were distinguished only by GMYC methodology (Fig 1 pattern D).

The BIN analysis recovered 56 distinct units (45 unpublished and 11 present in the BOLD database). In several cases the BIN delimitation was discordant with the morphological delimitation (Fig 2). Some species that were genetically very divergent were identified as a single OTU, such as *R*. *jaraguensis* & *R*. *kronei* & *R*. *langei*, *R*. *capitonia* & *R*. *microlepidogaster*, *R*. *cubataonis* & *R*. *lima* & *R*. *quadrensis*, *R*. *stellata* & *R*. *reisi* & *R*. *strigilata* & *Rineloricaria* sp. 4, and *R*. *zaina* & *R*. *anitae* & *R*. *anhanguapitan* & *R*. *maacki* & *R*. *tropeira* & *Rineloricaria* sp.5. Species such as *R*. *platyura*, *R*. cf. *wolfei*, *R*. *morrowi*, *R*. *heteroptera*, *R*. *fallax*, *R*. *lanceolata* and *R*. *aurata* were divided into more than one OTU (Fig 2).

In the GMYC analysis, the threshold time obtained was -4.26x10^-3^T, where T = time from present to the time of the root (Fig 2). Using this model, the results suggested the recognition of 70 putative species, 19 of which contained a single individual, and the confidence limits for the estimated number of species ranged from 44 to 83. The GMYC analysis confirmed the identity of 43 of 53 morphospecies. The species *R*. *cubataonis* and *R*. *lima* were identified as a single entity. The same occurred with *R*. *capitonia* and *R*. *microlepidogaster*, as well as with *R*. *kronei* and *R*. *langei*. Conversely, the species *R*. *platyura*, *R*. cf. *wolfei*, *R*. *morrowi* and *R*. *fallax* were divided in two OTUs each, while *R*. *lanceolata* and *R*. *aurata* had seven and six OTUs, respectively.

## Discussion

In our results, 41% of the delimitation of species within *Rineloricaria* were in accordance with genetic and morphological definitions (Fig 1 pattern A), which was close to the unified species concept proposed by de Queiroz [33]. Fifteen were undescribed species, from which 13 were recognized by the three independent analyses, demonstrating that DNA barcoding can help to identify new taxa in complex groups, as previously observed in *Starksia* [2], *Tetragonopterus* [34], *Neoplecostomus* [35], *Macrourus* [36], *Parapercis* [37] and other genera.

In 19 cases (Fig 1 pattern B), the morphospecies were not discriminated in BIN analysis; in six of these cases, the GMYC results were in accordance with the BIN determination, demonstrating that the GMYC analysis was more efficient than the BIN analysis for discriminating species of *Rineloricaria*, which were different from those found in other groups of organisms [9,38]. In all six cases in which both genetic analyses disagreed from the morphological identification, the morphospecies were genetically similar, and this similarity could be explained by the evolutionary history of these species as discussed below.

The GMYC analyses found more OTUs than the other methodologies and, although in the majority of cases corroborated the morphological identification, the GMYC indicated the existence of possible species complexes in *Rineloricaria*. Our results showed that the identification of an OTU is dependent on the analysis method, and, in *Rineloricaria*, the GMYC analysis was more efficient than the BIN analysis, which may be a sign that algorithms currently performed with BIN may suffer interference when employed in hyperdiverse groups.

### Finding the species complexes

As time passes in the speciation process, the boundaries between new species become increasingly evident [33]. However, at the beginning of this process (known as the grey zone sense [33]), the boundaries among species were hardly identified, making the species boundary very subjective and dependent on the concept of species [33]. The results obtained with *Rineloricaria* showed that the species limits do not appear in a fixed order; in some cases, the morphological limits appeared before the genetic limits (i.e., a single locus analysis), as observed in *R*. *kronei* and *R*. *langei*. However, the genetic limit in other cases preceded the morphological, such as in *R*. *aurata* and *R*. *lanceolata*.

Currently, DNA barcoding techniques are used as an additional methodology to help with species delimitation in Neotropical fishes and to support new species descriptions (e.g., [34,35,39]). Moreover, the barcoding techniques are frequently helpful for highlighting species complexes [3,40] and could be an excellent start to traditional taxonomy work [38].

### Cryptic species

The great genetic divergence between the lineages that are morphologically indistinct (patterns C and D) is evidence of cryptic species [3,40,41], and this is the case found in the morphospecies *Rineloricaria* cf. *wolfei*, *R*. *platyura*, *R*. *morrowi* and *R*. *fallax*, with each one have two lineages with genetic differentiation ranging between 1.1% to 2.4%. In extreme cases of cryptic species, as with *R*. *lanceolata* and *R*. *aurata* that have seven and six OTUs, respectively, genetic differentiation between lineages varies from 1.3% to 8.5%. The variation found among lineages of these morphospecies was larger than the variation found between distinct species of *Rineloricaria*, and this fact could indicate that the divergence time between lineages could be sufficient to establish reproductive isolation. However, given that the lineage present in each of these morphospecies does not occur sympatrically in the ecoregions (sensu [42]), the variation among the lineages could be due to mutation accumulation over time in geographical isolation and not necessarily due to reproductive incompatibility [38]. The exception is the *R*. *heteroptera* morphospecies that presents two OTUs with more than 4% of K2P distance; these OTUs are present in the same ecoregion (both OTUs are in Rio Negro on the Amazon river basin). Therefore, the OTUs present in *R*. *heteroptera* morphospecies probably cannot interchange genes due reproductive isolation, as discussed by Kekkonen et al. [38], which characterized these OTUs as distinct species by biological concepts [43]. Moreover, the OTUs found in *R*. *heteroptera* morphospecies are not assembled in a monophyletic cluster, which reinforces the hypothesis of a species complex. The morphotype found in *R*. *heteroptera* can be caused by convergences related to selective pressures driven by similar ecological conditions, as well as in species of *Astyanax* from Central America [44].

### Low genetic divergences among species

Our results revealed the following species with low genetic divergence (corresponding to pattern B): *R*. *lima* & *R*. *cubataonis* (0.8% of genetic divergence), *R*. *microlepidogaster* & *R*. *capitonia* (0.8% of genetic divergence) and *R*. *langei* & *R*. *kronei*, which did not present genetic differentiation, probably because these species did not yet reach the reciprocal monophyly. The genetic patterns found in *R*. *lima* & *R*. *cubataonis* and *R*. *microlepidogaster* & *R*. *capitonia* are very similar. In both cases, the level of genetic divergence is equivalent to that found among populations of a single species. These morphospecies are isolated in distinct, disconnected ecoregions in the same river basin. A similar example was found in other fish groups, such as *Parodontidae* [41], indicating a recent vicariance process followed by a fast morphological differentiation.

The DNA barcoding techniques were not effective in discriminating between *R*. *kronei* and *R*. *langei*, probably because these species did not reach the reciprocal monophyletic. A similar situation was found in the genus *Ixinandria* by Rodriguez et al. [4], who tried to define the boundaries between *I*. *steinbachi* and *I*. *montebelloi* using morphological and genetic characters and discovered that this species was not reciprocally monophyletic. The difference in our results is that, in Rodriguez et al. [4], they considered the morphologic distinction insufficient to validate the two species and opted for synonymy, while we are of the opinion that the morphological differences between *R*. *kronei* and *R*. *langei* are sufficient to discriminate between them. Moreover, this species occupies different types of habitats [17,45] with specific morphological adaptations in each environment [14]. Therefore, another explanation is necessary to solve the issue of *R*. *langei* and *R*. *kronei*. It is tempting to suggest extremely recent speciation, as probably occurred with the species *R*. *lima* & *R*. *cubataonis* and *R*. *microlepidogaster* & *R*. *capitonia*. However, unlike these species, *R*. *kronei* was collected in sympatry with *R*. *langei*, and *R*. *langei* was collected in several unconnected river systems. Accordingly, it is unlikely that recent vicariance processes have been responsible for the divergence among them because several and successive geodispersal processes would be necessary in a very short time to obtain this scenario.

Furthermore, our results regarding *R*. *langei* and *R*. *kronei* were very similar to those found by Kashiwagi et al. [46], who studied two morphologically distinct manta ray species (*Manta alfredi* and *M*. *birostris*). However, Kashiwagi and colleagues, using nuclear markers, discovered that the manta ray species were distinguished by their nuclear DNA and concluded that a mitochondrial introgression occurred by hybridization among the manta ray species. The genetic patterns found in *R*. *langei* and *R*. *kronei* could be explained by mitochondrial introgression. To confirm this hypothesis, further investigation is needed using other molecular markers such as nuclear genes and cytogenetic characterizations.

DNA barcoding using an integrative approach of molecular and taxonomic methods, mixing the BINs and the GMYC model, was very efficient for delimiting species of *Rineloricaria*, mainly in groups with low morphological variation (i.e., cryptic species). The recognition of different genetic structures in groups with very similar morphology has exposed a common pattern across the tree of eukaryotic life, and it is observed particularly often in species-rich genera, as observed in skipper butterfly [47], hammerhead sharks [48], arthropods [49], Australian fishes [22] and neotropical fishes [35,41,50].

Here, we adopted some OTU hypotheses not supported by more than one methodology, as in the patterns B, C and D. However, the fact that one analysis pointed to the existence of a new entity encourages the pursuit of studies with different methodologies to build a more robust hypothesis. Moreover, if one of the OTUs found here was not a distinct biological species (sense [43]), it does not mean that it should not be protected. Even if the OTUs were reproductively compatible, the deep genetic diversity of the lineage of Rineloricaria showed that morphospecies were widely distributed and were fragmented into several local lineages. Thus, a great effort is needed to preserve the diversity of the genus and maintain local lineages. The integrative methods have broad implications in unveiling species diversity, with large implications on conservation politics and the delineation of preservation areas.

## Supporting Information

S1 Fig The within-species distribution is normalized to reduce bias in sampling at the species level. The table below summarizes this distribution, while the histogram plots the distribution of normalized divergence for species (pink) against the genus divergences (green).
(TIF)

S2 Fig Detail of the Bayesian phylogenetic tree of Rineloricaria obtained with COI data: The red line shows the threshold found in the GMYC analysis. The blue bars are presents in the nodes with more than 95% of posterior probability and represent the variance rate of the node.
(JPG)

S1 File Rineloricaria COI sequences. (FAS)

S1 Table Samples data bank. (XLSX)

S2 Table Summary of the genetic variation in each morphospecies. (XLS)
