# Supplementary figures and images for: Using Different Methods to Access the Difficult Task of Delimiting Species in a Complex Neotropical Hyperdiverse Group
Source: PLoS One. 2015 Sep 2;10(9):e0135075. doi: 10.1371/journal.pone.0135075 (PMC4557985; doi:10.1371/journal.pone.0135075)

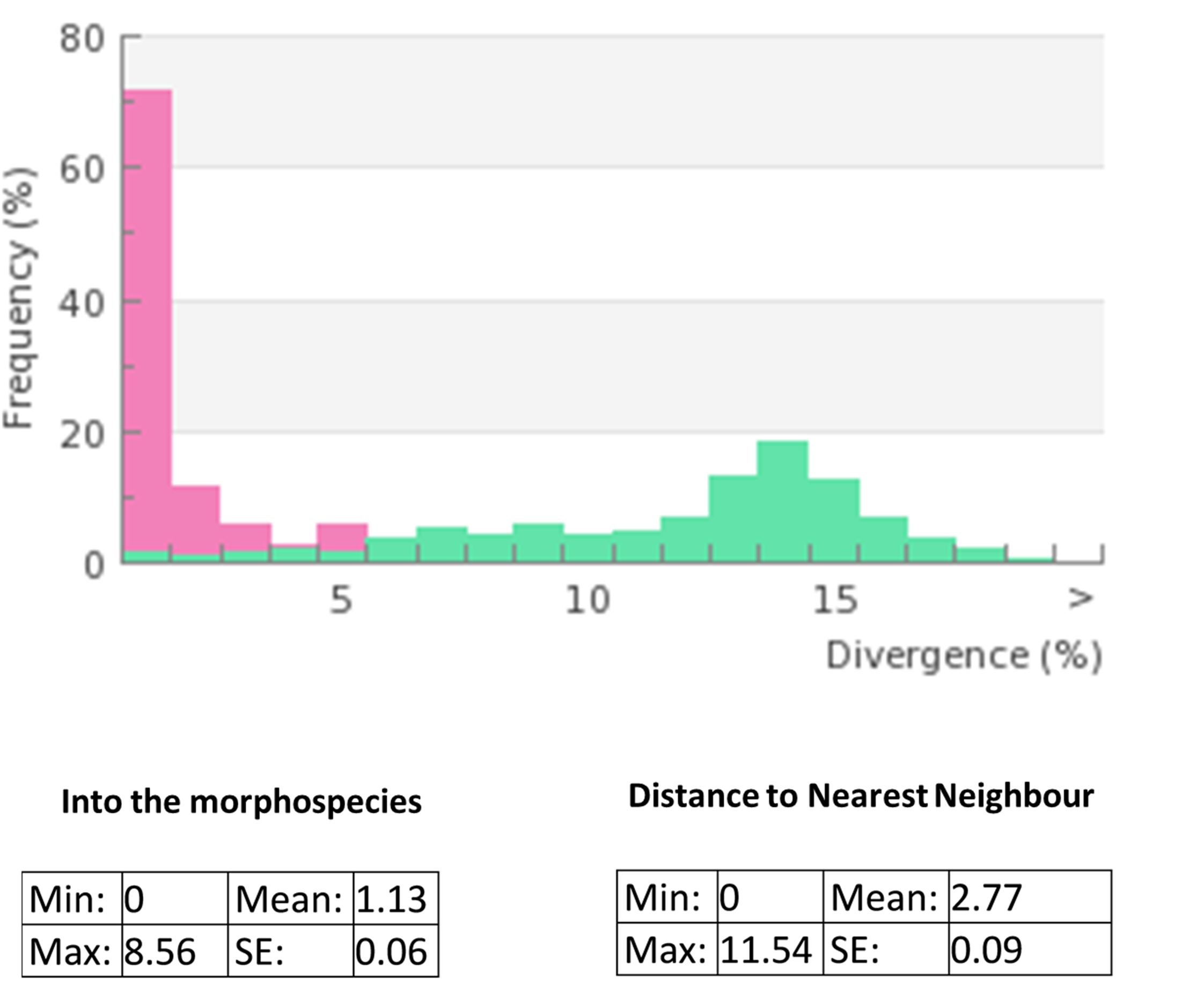

Supplement: S1 Fig — The table below summarizes this distribution, while the histogram plots the distribution of normalized divergence for species (pink) against the genus divergences (green). (TIF) [file pone.0135075.s001.tif]

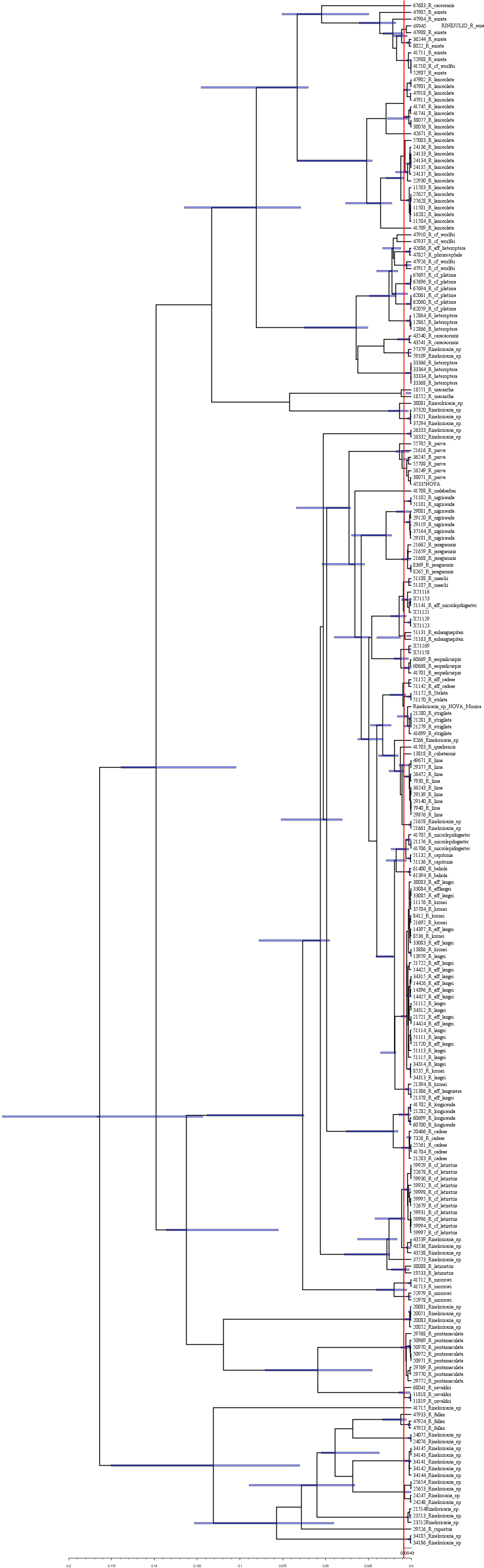

Supplement: S2 Fig — The blue bars are presents in the nodes with more than 95% of posterior probability and represent the variance rate of the node. (JPG) [file pone.0135075.s002.jpg]
